# Protein KIC5 is a novel regulator of artemisinin stress response in the malaria parasite *Plasmodium falciparum*

**DOI:** 10.1038/s41598-023-27417-6

**Published:** 2023-01-09

**Authors:** Caroline F. Simmons, Justin Gibbons, Min Zhang, Jenna Oberstaller, Camilla Valente Pires, Debora Casandra, Chengqi Wang, Andreas Seyfang, Thomas D. Otto, Julian C. Rayner, John H. Adams

**Affiliations:** 1 Center for Global Health and Infectious Diseases Research and USF Genomics Program, College of Public Health, University of South Florida, Tampa, FL USA; 2 Department of Molecular Medicine, Morsani College of Medicine, University of South Florida, Tampa, FL USA; 3 Institute for Infection, Immunity, and Inflammation, College of Medical, Veterinary and Life Sciences, University of Glasgow, Glasgow, UK; 4 Cambridge Institute for Medical Research, University of Cambridge, Cambridge Biomedical Campus, Cambridge, Cambridgeshire UK

**Keywords:** Parasite genetics, Parasite genomics, Malaria

## Abstract

Artemisinin combination therapies (ACTs) have led to a significant decrease in *Plasmodium falciparum* malaria mortality. This progress is now threatened by emerging artemisinin resistance (ART-R) linked originally in SE Asia to polymorphisms in the Kelch propeller protein (K13) and more recently to several other seemingly unrelated genetic mutations. To better understand the parasite response to ART, we are characterizing a *P. falciparum* mutant with altered sensitivity to ART that was created via *piggyBac* transposon mutagenesis. The transposon inserted near the putative transcription start site of a gene defined as a “*Plasmodium-*conserved gene of unknown function,” now functionally linked to K13 as the Kelch13 Interacting Candidate 5 protein (KIC5). Phenotype analysis of the KIC5 mutant during intraerythrocytic asexual development identified transcriptional changes associated with DNA stress response and altered mitochondrial metabolism, linking dysregulation of the KIC5 gene to the parasite’s ability to respond to ART exposure. Through characterization of the KIC5 transcriptome, we hypothesize that this gene may be essential under ART exposure to manage gene expression of the wild-type stress response at early ring stage, thereby providing a better understanding of the parasite’s processes that can alter ART sensitivity.

## Introduction

Malaria is a global mosquito-borne parasitic disease that in 2020 caused clinical illness in ~ 241 million people and killed ~ 627,000, the majority of which were children less than 5 years old^1^. Treatment and control of malaria cases particularly by *Plasmodium falciparum* depends on artemisinin (ART) class antimalarials in combination with longer acting non-artemisinin partner drugs. These potent artemisinin combination therapies (ACTs), along with preventative methods and rapid diagnostic tests, has led to an overall 19.7% decline in malaria cases from 2010 to 2018 worldwide^1^ and a significant decrease in mortality from *P. falciparum* infections. The potent and fast acting drug is toxic to both the asexual and sexual stages of the parasite with a promiscuous mechanism of activity that is still not completely defined^2^. The primary mechanisms of ART antimalarial cytotoxicity are attributed to disruptions to hemoglobin uptake and digestion, protein synthesis and degradation, and cell cycle regulation^3^. Other molecular factors have been, and continue to be, linked to ART activity, including disruption of mitochondrial function through membrane potential depolarization^4^. These findings display the increasing need to comprehensively annotate the pathways and molecular components targeted by ART to assist in the global effort of developing novel and potent malaria treatments. Increased ART resistance (ART-R) first observed in SE Asia and emerging in Africa increases the need for a greater understanding of antimalarial activity in the parasite since the lack of efficacy of ART facilitates emergence of resistance for partner drugs, leading to the lost efficacy of ACTs as a reliable treatment to cure and control malaria^1^.

The ART-R resistance phenotype is associated with delayed clearance time post-treatment and was first linked to polymorphisms in *P. falciparum’s* Kelch propeller protein (*kelch13,* K13), which is now considered a primary molecular marker for clinical artemisinin resistance in the Greater Mekong Region^5,6^. Mechanisms behind K13-mediated resistance include reduced activation of artemisinin antimalarials, enhanced parasite ability to mitigate damaged proteins, increased vesicular cell trafficking to fortify cell membrane from damage, and alteration of erythrocytic cell cycle progression conferring an elongated ring stage, the least metabolically active stage of the parasite^7^. Our recent study and others revealed that *P. falciparum* appears to exploit its innate fever response mechanism to augment resistance to artemisinin, utilizing essential and conserved pathways linked to protein-folding, heat shock, proteasome activity, and apicoplast-derived isoprenoid biosynthesis^7,8^. Unfortunately, as the resistance phenotype continues to spread globally, there has been evidence of artemisinin resistance in *P. falciparum* conferred by genes other than K13, including coronin and UBP-1^9–11^. Though mutations to these genes have not yet been widely detected in clinical isolates, the ability of the parasite to become resistant to artemisinin via mechanisms other than K13 mutations stresses the need for additional molecular analysis and surveillance of non-K13 mediated changes to ART activity^9,10^.

To improve our understanding of the genetics of *P. falciparum* drug resistance, our laboratory developed a forward genetic method in *P. falciparum* by creating random single insertion disruptions with the *piggyBac* transposon^12^. Via chemogenomic profiling of *piggyBac* mutants, we can elucidate genotype–phenotype associations linked to artemisinin (ART) as well as other antimalarial drugs and inhibitors. Our initial chemogenomics screen identified an “Artemisinin Sensitivity Cluster”, a set of 7 *piggyBac* mutant clones with increased sensitivity to artemisinin compounds, including a mutant of K13^13^. This K13 *piggyBac-*mutant exhibits altered K13 gene expression, leading to significant dysregulation of DNA replication and repair pathways, consistent with the role of K13 as a stress response regulator^14^. In the current study, we are characterizing another clone from this cluster that has a single *piggyBac* insertion near the putative transcription start site of PF3D7_1138700. Annotated as a dispensable *Plasmodium*-conserved gene of unknown function, it has been functionally linked to K13 as a “Kelch13 Interacting Candidate 5” (KIC5) of the K13 endocytosis cluster in modifying parasite uptake of host cell cytosol, and subsequently hemoglobin, altering the rate of hemoglobin digestion and ART activation^15,16^. In the same study, KIC5 was found to co-localize with K13, and knock sideways studies of KIC5 decreased *P. falciparum* sensitivity to ART and delayed IDC growth^16^. Given the recently annotated interactions of KIC5 with K13 and its increased sensitivity to ART, we decided to examine the KIC5 *piggyBac* mutant by transcriptional profiling to elucidate the changes associated with its ART-S phenotype. Here, we find transcriptional changes associated with this mutant identify KIC5 as a key early regulator of *P. falciparum’s* response to artemisinin.

## Results

### Characteristics of the KIC5 mutant

**Figure 1:**
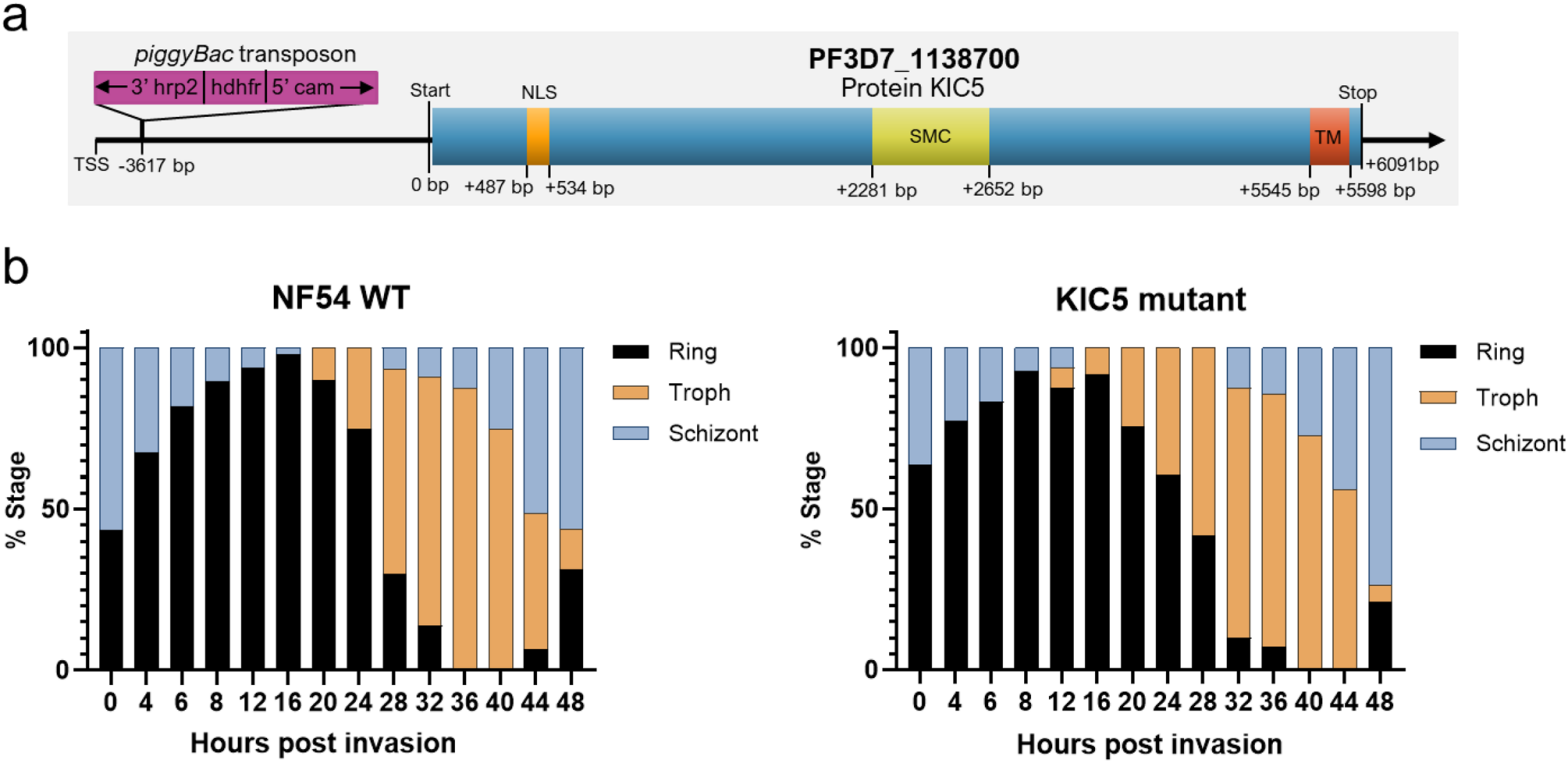
Characteristics of the KIC5 mutant. (**a**) The *piggyBac* transposon insertion in the KIC5 mutant lies within the 5′ UTR of the KIC5 gene, specifically 10 bp+ of the annotated TSS. (**b**) Analysis of the percentages of ring, trophozoite, and schizont during the 48-h intraerythrocytic cycle progression in wild-type NF54 (left) and KIC5 mutant (right). Results were obtained via microscopic analysis of synchronized cultures with two bio-replicates. Fisher’s Exact Test was performed, determining no significant differences between the amount of ring, trophozoite, and schizont microscopy cell counts per timepoint for the KIC5 mutant vs NF54 (Supplementary Table S1). Graphs created in GraphPad Prism 9 and statistics performed in R. *NLS* nuclear localization signal, *SMC* structural maintenance of chromosome domain, *TM* transmembrane domain.

The KIC5 mutant clone contains a *piggyBac* transposon insertion in the five prime untranslated region of PF3D7_1138700, located 10 bp downstream of the annotated transcription start site (Fig. 1a)^17,18^. Morphological analyses of the mutant compared to the isogenic wild-type NF54 through intraerythrocytic development cycle progression (rings, trophozoites, schizonts) every 4 h post merozoite invasion of RBCs revealed no significant changes (Fisher’s Exact Test, p-value ≥ 0.05) (Fig. 1b, Supplementary Table S1). Additionally, we validated via microscopy the morphological stages of parasite samples used for RNAseq analysis (Supplementary Fig. 1). Again, we confirmed no significant difference (Fisher’s Exact Test, p-value ≥ 0.05) in IDC stage for the samples collected for this study (Supplementary Table S2).

Previously, chemogenomic profiling found the drug response of this KIC5 mutant to be highly correlated to a *piggyBac* mutant of K13, similarly containing a 5′ UTR *piggyBac* transposon insertion^13^. Compared to its isogenic wild-type NF54 parent clone, the KIC5 mutant had increased sensitivity to dihydroartemisinin (fold change (FC) GI50: 0.20) and artemisinin (FC GI50: 0.29) based on differences in relative growth (Supplementary Fig. 2a)^13^. Additionally, the KIC5 mutant was found to have increased sensitivity to benzimidazole (FC GI50: 0.22), sinefungin (FC GI50: 0.38), and primaquine (FC GI50: 0.25), which all had highly correlated drug response profiles to that of artemisinin^13^.

Little is known about the function of KIC5 in *P. falciparum* except as a K13 endocytosis complex interacting candidate^16^. KIC5 has been previously reported to be associated with *response to drug* biological process (GO:0042493) and *nucleus* cellular component (GO:0005634)^17,18^. Additionally, KIC5 was predicted to interact with protein KIC6 (PF3D7_0609700), a putative chromodomain-helicase-DNA-binding protein 1 homolog (PF3D7_1023900), and merozoite surface protein 2 (PF3D7_0206800)^19^. These interacting partners are supported in part with bioinformatic analysis of KIC5 that predicted a nuclear localization signal at the N-terminus, presence of a putative Smc domain (Chromosome segregation ATPase), and a DNA double-strand break repair ATPase Rad50 domain, linking KIC5 to the parasite’s DNA metabolism in (Fig. 1a, Supplementary Fig. 2b,c).

### KIC5 disruption significantly alters expression patterns in *P. falciparum*

To confirm transcriptional alignment of our NF54 and KIC5 mutant samples, we analyzed correlation between the two clones for the five timepoints tested in this study (6 h post merozoite invasion, 12 h, 24 h, 36 h, and 48 h) (Supplementary Fig. 3). FPKM gene expression values were used to determine Spearman correlation coefficients corresponding to each timepoint (Supplementary Table S3), with correlation values > 0.9 across the identical timepoints between NF54 and the KIC5 (Supplementary Table S5). Additionally, we analyzed the Spearman correlation between NF54 and the KIC5 mutant of several housekeeping pathways to determine transcriptional matching at the metabolic level (Supplementary Fig. 4). Analysis of FPKM expression of genes associated with translation, transcription, DNA replication and repair, and the proteasome showed high correlation values (> 0.8) between NF54 and KIC5 mutant for identical timepoints, supporting global transcriptomic similarity between our samples (Supplementary Tables S6 and S7). Lastly, we analyzed correlation of the NF54 and KIC5 mutant datasets from this study with previously published NF54 expression data from Gibbons et al. (Supplementary Fig. 5)^14^. Again, high correlation is seen between our NF54 and KIC5 mutant datasets and the previously published NF54 transcriptome at identical timepoints, providing additional support for transcriptional alignment of our samples (Supplementary Table S8).

**Figure 2:**
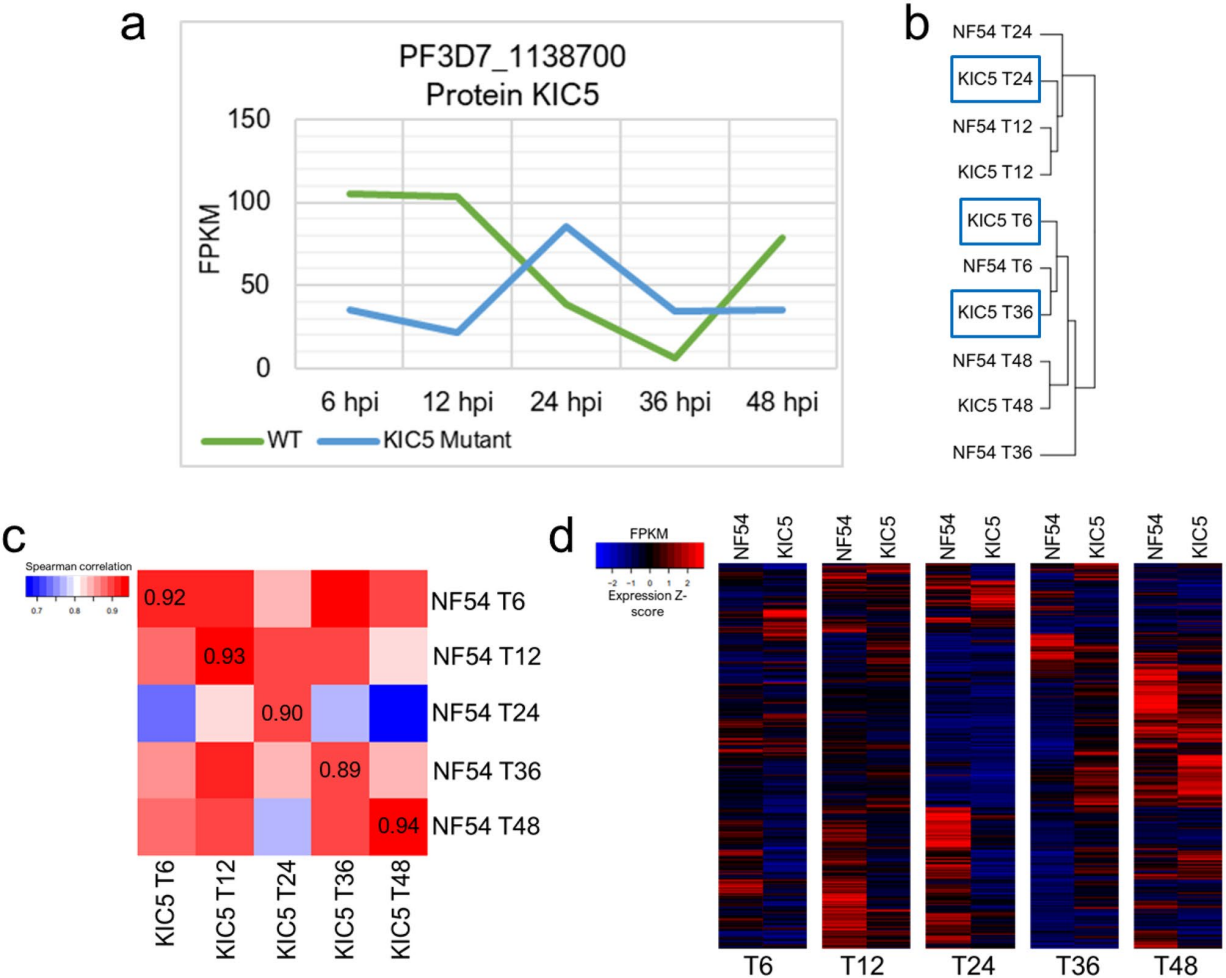
The KIC5 mutant has an altered transcriptome compared to the isogenic wild-type NF54. (**a**) Expression of the KIC5 gene across the 5 timepoints analyzed in this study show peak expression of the gene change from early ring in NF54 (green line) to early trophozoite stage in the mutant (blue line). Results are shown in fragments per kilobase per million mapped reads (FPKM) averaged across two bio-replicates (Supplementary Table S3). (**b**) Dendrogram of WT and KIC5 mutant timepoint expression correlations show alternate correlations of KIC5 6 hpi, 24 hpi, and 36 hpi. Dendrogram obtained via hierarchal clustering of NF54 and KIC5 mutant FPKM expression from heatmap.2 function in R Studio. (**c**) Spearman correlation heatmap of KIC5 mutant timepoints to NF54 timepoints (Spearman correlation values in Supplementary Table S5). (**d**) Heatmap comparison of gene expression in WT vs the KIC5 mutant across timepoints analyzed in this study show that gene expression is altered in the KIC5 mutant. Shown in Expression Z-score of gene FPKM values (Supplementary Table S3).

Significant upregulation of KIC5 occurred in the mutant at 24 h post merozoite invasion (hpi), compared to NF54 (Fig. 2a, p-value = 0.0332), that resulted in peak KIC5 expression shifting from early ring stage in the WT (6 hpi) to early trophozoite stage (24 hpi) in the mutant. Additionally, genes flanking KIC5 lacked significant dysregulation throughout the IDC (Supplementary Fig. 6, p-value ≥ 0.05). Hierarchal clustering of NF54 and KIC5 mutant global FPKM expression for each timepoint was performed, revealing correlated expression between the two clones at only 12 hpi and 48 hpi, supplementing Spearman correlation statistical analysis (Fig. 2b, Supplementary Table S5). NF54 parent clone exhibits transcriptional changes from ring to trophozoite stages, with metabolic activities such as DNA replication, translation, transcription, hemoglobin digestion, and trafficking being upregulated in trophozoite stage^20–22^. In the mutant, we observed gene dysregulation at 6 hpi, 24 hpi, and 36 hpi, with 24 hpi expression closely correlating to NF54 at both 12 hpi and 24 hpi (Fig. 2c, Supplementary Fig. 3, Supplementary Table S5). Overall, we observed differences in FPKM values across all timepoints sampled for the KIC5 mutant, confirming global gene dysregulation (Fig. 2d, Supplementary Table S3).

### Gene ontology enrichment and clustering of KIC5 mutant differential expression confirms pathway co-dysregulation patterns

**Figure 3:**
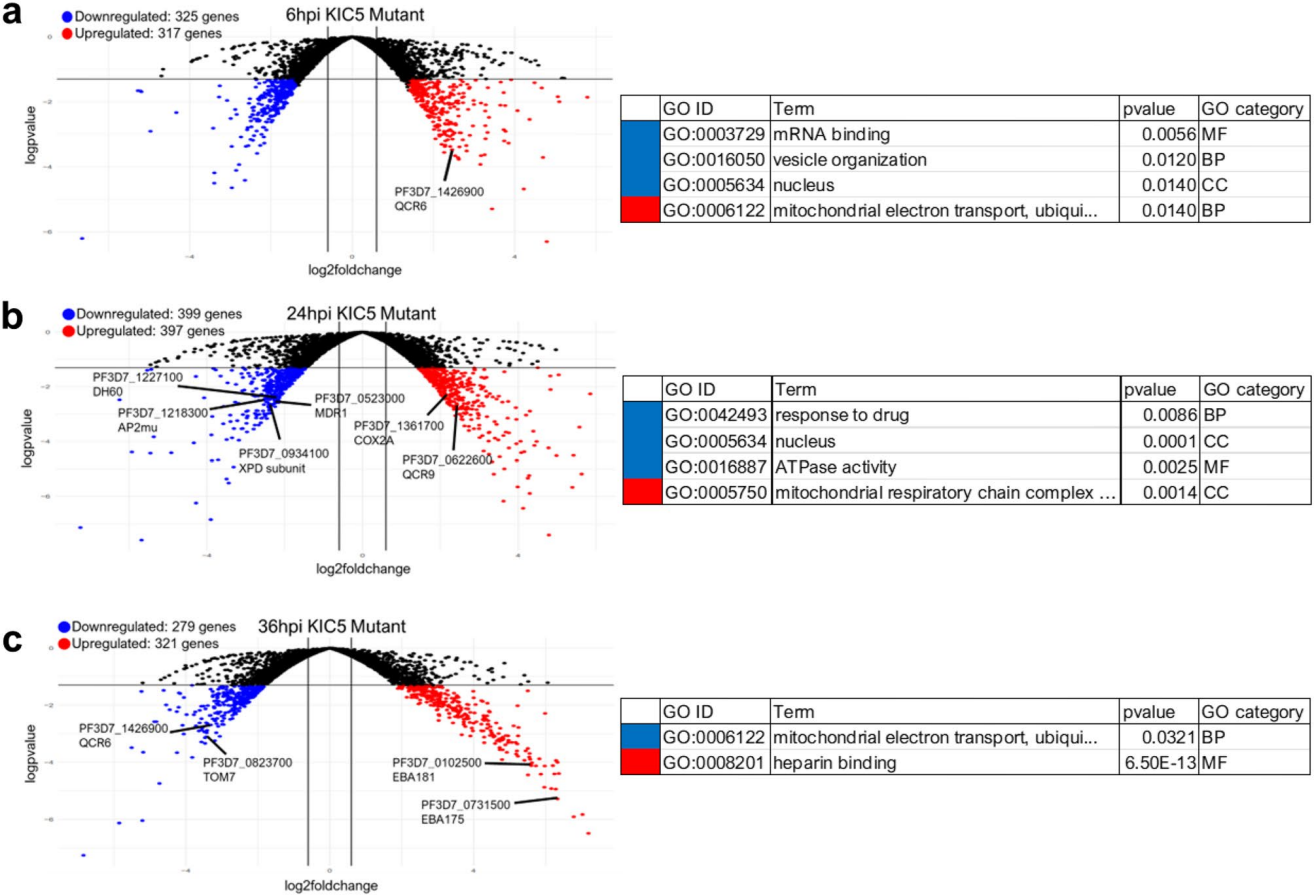
Differentially expressed genes and gene ontologies in the KIC5 mutant at 6 hpi, 24 hpi, and 36 hpi. Volcano plots showing differential gene expression at (**a**) 6 hpi [downregulated: 325 genes, upregulated: 317 genes], (**b**) 24 hpi [downregulated: 399 genes, upregulated: 397 genes], and (**c**) 36 hpi [downregulated: 279 genes, upregulated: 321 genes] (Supplementary Table S4). Compared to 12 hpi and 48 hpi, there are an increased number of DE genes at these three chosen timepoints. For each timepoint shown, R package pfGO (v 1.1) was used to perform gene ontology enrichment. Select upregulated and downregulated gene ontologies related to GO cellular compartment (CC), GO molecular function (MF), or GO biological process (BP) are displayed (Supplementary Table S9). Blue: downregulated genes, red: upregulated genes. Results plotted with log2-fold change (KIC5 mutant/NF54 WT) vs log2 p-value. FDR corrected p-value < 0.05; log2-fold change upregulated > 1.4, downregulated < − 1.4.

The number of significantly differentially expressed genes (p-value < 0.05) at 6 hpi (upregulated: 317 genes; downregulated: 325 genes), 24 hpi (upregulated: 397 genes; downregulated: 399 genes), and 36 hpi (upregulated: 321 genes; downregulated: 279 genes) varied noticeably from differentially expressed genes at 12 hpi (upregulated: 140 genes; downregulated: 74 genes) and 48 hpi (upregulated: 31 genes; downregulated: 87 genes) (Fig. 3a–c, Supplementary Table S4). Gene ontology enrichment analysis was performed using the pfGO package v 1.0 on these significantly upregulated and downregulated gene sets at 6 hpi, 24 hpi, and 36 hpi to further identify the pathways associated with differential expression in the mutant (Supplementary Tables S9 and S10)^23^.

Dysregulation of genes linked to *response to drug* (GO:0042493) occurs at timepoints where KIC5 expression is dysregulated, 6 hpi and 24 hpi (Fig. 3b, Supplementary Table S9). Dysregulated genes at 24 hpi associated with this GO term include ATP6, MRP1, MDR1, ATP4, AP2-MU, and MRP2 (Supplementary Table S10). Genes linked to altered ART response downregulated at the least ART-S stage, 6 hpi, included MRP1, MRP2, Eps15-like protein, along with three ART sensitivity cluster genes (autophagy-related protein 7 putative [PF3D7_1126100], conserved *Plasmodium* protein, unknown function [PF3D7_1136600], and serine/threonine protein kinase, FIKK family [PF3D7_0902200]) (Supplementary Table S10)^13,24^. Differentially expressed genes linked to response to ART, combined with dysregulation of genes associated with *response to drug* GO at 24 hpi, points to a potential loss-of-function associated with ART response pathways at 6 hpi and 24 hpi.

Recent studies have shown how damage sensing processes, antioxidant properties, and altered electron transport chain activities of the mitochondrial metabolism may augment the ability of K13 mutants to survive dihydroartemisinin treatment in early ring stage^25,26^. Further gene ontology enrichment showed changes to the expression of genes linked to mitochondrial metabolism, potentially linking KIC5 to these pathways. The following GO terms were enriched in our dataset at 6 hpi, 24 hpi, and/or 36 hpi: *mitochondrial electron transport, ubiquinol to cytochrome c* (GO:0006122), *mitochondrial outer membrane translocase complex* (GO:0005742), *mitochondrial respiratory chain complex III* (GO:0005750), and *mitochondrial respiratory chain complex IV* (GO:0005751) (Supplementary Tables S9 and S10). These mitochondrial electron transport chain pathways were upregulated at 6 hpi and 24 hpi, and subsequently downregulated at 36 hpi, thus demonstrating a transcriptional disruption of electron transport chain activity in the mutant (Fig. 3a–c) (Supplementary Tables S9 and S10).

GO enrichment analysis further identified *nucleus* (GO:0005634) as a top GO cellular compartment downregulated at 6 hpi and 24 hpi (Fig. 3a,b, Supplementary Table S9). Genes linked to this GO term in our dataset represented DNA replication pathways, proteasome metabolism, mRNA processing, transcription factors, genes with ATPase activity and various other DNA-binding proteins or proteins of unknown function (Supplementary Tables S9 and S10). As artemisinin compounds have been shown to induce DNA damage in *P. falciparum*, responses to DNA damage play a critical role in the parasite’s ability to mitigate ART exposure^27^. This dysregulation of a wide array of nuclear localized genes combined with nucleus-associated domains suggests a link between KIC5 and nuclear metabolism.

**Figure 4:**
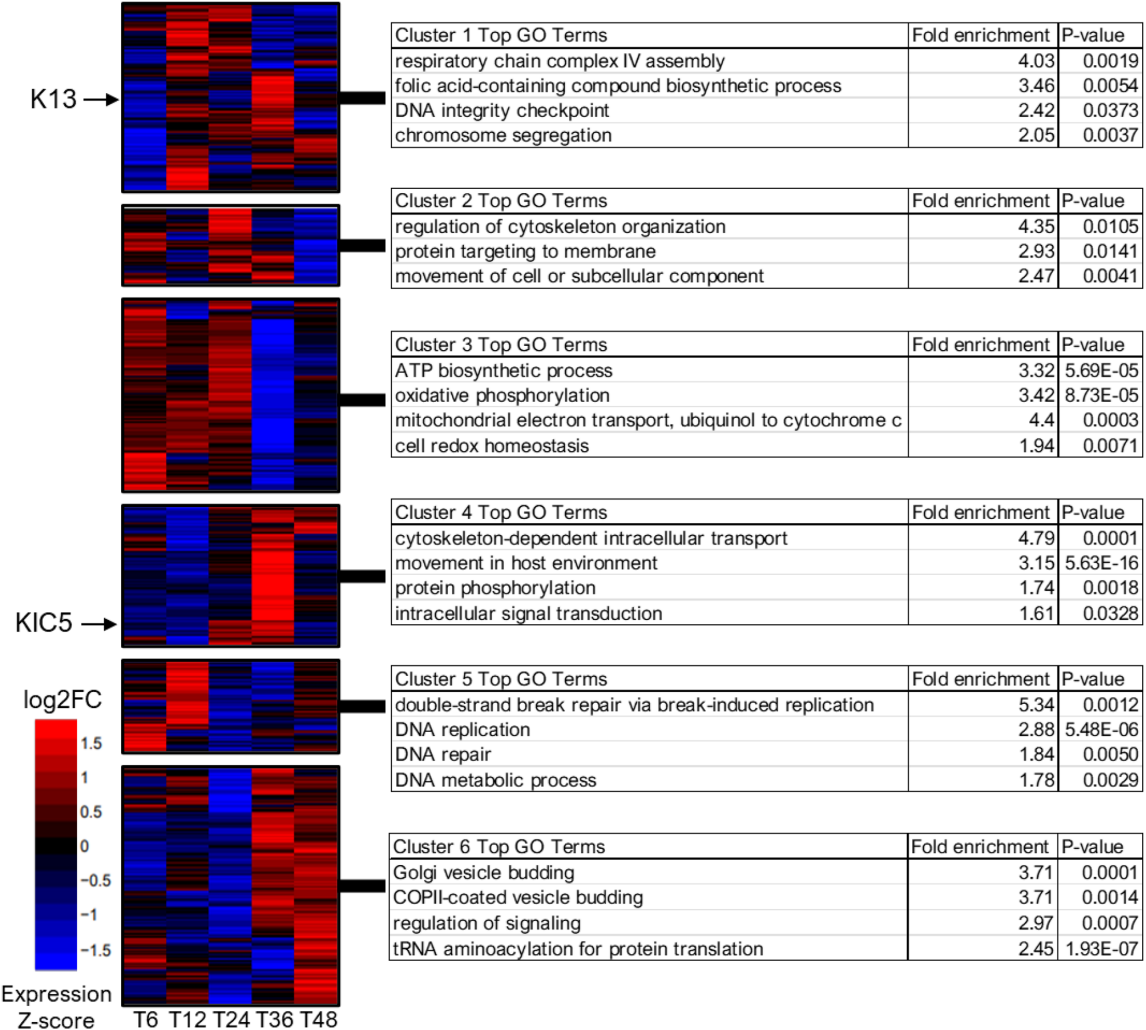
Clustering of dysregulation genes across the KIC5 transcriptome. Cluster heatmap of KIC5 mutant log2-fold change (log2FC) values. Bioinformatic analysis to determine optimal clustering identified 6 gene clusters with similar differential expression patterns in the KIC5 mutant clone compared to the isogenic wild-type NF54 clone (Supplementary Fig. 7, Supplementary Table S11). The location of protein KIC5 and K13 in the heatmap are indicated with arrows. Heatmap displayed as Expression Z-score of log2-fold change (KIC5 mutant/NF54) values. Top GO biological processes of each cluster are shown in the corresponding tables, analyzed via PlasmoDB gene ontology enrichment tool (Supplementary Table S12). Significant p-value < 0.05.

We next investigated which molecular pathways were co-dysregulated in response to KIC5 disruption. After determining the statistically optimal number of gene expression clusters based on fold change expression (Supplementary Fig. 7), we created a global expression heatmap of six gene clusters to perform a gene ontology enrichment for biological processes (Fig. 4, Supplementary Tables S11 and S12). We identified a cluster of genes (cluster 3) enriched in oxidative phosphorylation, mitochondrial electron transport ubiquinol to cytochrome *c*, ATP biosynthetic process, and cell redox homeostasis, upregulated at 6–24 hpi and downregulated at 36 hpi. Consistent with our analysis of significantly differentially expressed genes, co-dysregulation of these pathways reflects broad dysregulation of mitochondrial electron transport chain activity in the KIC5 mutant.

Additionally, we identified a cluster of genes (cluster 5) found to be highly enriched in biological processes such as *DNA replication*, *double-strand break repair *via* break-induced replication*, and *DNA repair* (Fig. 4). When analyzing IDC expression, cluster 5 genes were downregulated at 24 hpi compared to the other timepoints. This finding further compliments our GO enrichment of differentially expressed genes at 6 hpi, 24 hpi, and 36 hpi and supports our hypothesis that genes linked to nuclear metabolism are differentially regulated in the mutant in response to KIC5 disruption.

### Altered expression of the mitochondrial respiratory chain complex in the KIC5 mutant supports the link between altered ART response and mitochondrial metabolism

**Figure 5:**
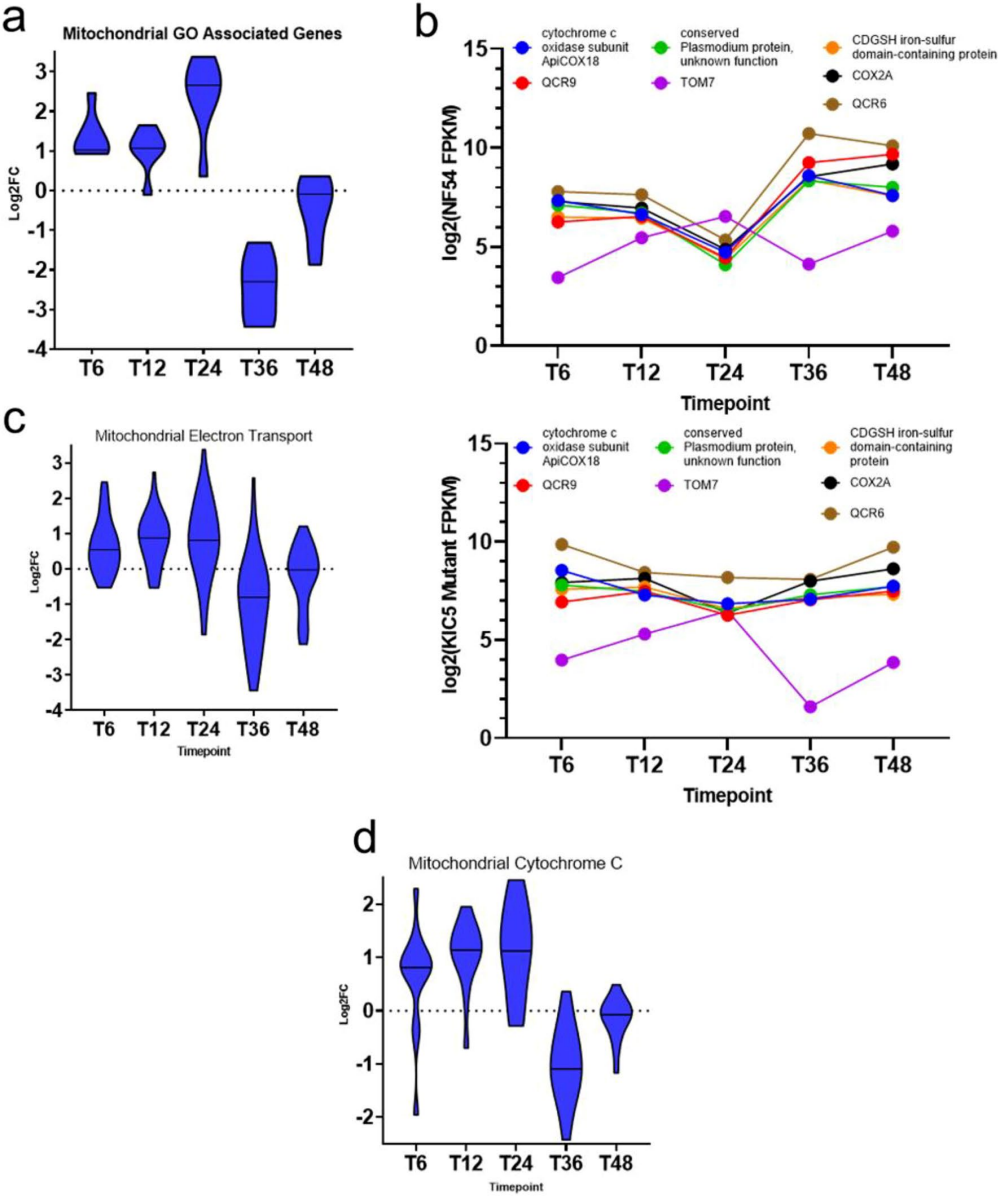
KIC5 disruption affects mitochondrial metabolism in *P. falciparum.* (**a**) Fold change analysis via violin plot shows upregulation of genes associated with top DE mitochondrial gene ontologies at 6–24 hpi (Supplementary Data file S10). Downregulation is seen at 36 hpi. Fold changes displayed as log2foldchange (log2FC; KIC5 mutant/NF54), and plots made in GraphPad Prism 9. (**b**) The expression patterns of several mitochondrial metabolic genes were found to be altered in the KIC5 mutant (right panel) from NF54 (left panel). Gene expression patterns plotted via log2(NF54FPKM) or log2(MutantFPKM) across five timepoints sampled in this study. Graphs created on GraphPad Prism 9. Using the Malaria Parasite Metabolic Pathways database, fold change analysis of genes involved in mitochondrial electron transport (**c**) and cytochrome *c* pathways (**d**) were found to have altered expression in the KIC5 mutant (Supplementary Table S13). Analysis identified an overall pattern of upregulation during early ring to early trophozoite stage and a noticeable downregulation at late trophozoite stage of these pathways. Dysregulation of mitochondrial pathways provides an additional link to altered ART-S and altered mitochondrial activity. Violin plotted using log2foldchange (KIC5 mutant/NF54) and created in GraphPad Prism 9.

To identify the mitochondrial pathways dysregulated in the KIC5 mutant, we analyzed the top differentially expressed genes of the mitochondrial metabolism. These included: TOM7 representing mitochondrial outer membrane translocase complex; QCR6; QCR9; a conserved *Plasmodium* protein unknown function (PF3D7_0817800) representing mitochondrial respiratory chain complex III; cytochrome *c* oxidase subunit ApiCOX18, putative; CDGSH iron–sulfur domain-containing protein, putative; and COX2A representing the mitochondrial respiratory chain complex IV (Table 1, Supplementary Table S10). These genes were upregulated at 24 hpi in the mutant, similar to KIC5 peak expression, with a noticeable downregulation at 36 hpi (Fig. 5a). Subsequently, we observed co-dysregulation of these genes in the mutant (Fig. 5b), however, co-dysregulation was not observed with TOM7 of the mitochondrial outer membrane translocase complex (mitochondrial import receptor subunit TOM7, putative [PF3D7_0823700]). As this gene is a mitochondrial gene not associated with the respiratory chain complex, our data points to specific altered activity of the mitochondrial respiratory chain complex as opposed to mitochondrial activity in general^28^. Further supporting this deduction is the significant dysregulation of QCR6 (cytochrome *b*–*c*1 complex subunit 6, putative [PF3D7_1426900]). Considered essential for the IDC and associated with mitochondrial respiratory chain complex III, specifically electron transport ubiquinol to cytochrome *c*, we found QCR6 to be significantly differentially expressed at 6 hpi (p-value = 0.0003), 24 hpi, (p-value = 6.53E−06) and 36 hpi (p-value = 0.0008) (Supplementary Fig. 8, Supplementary Table S10)^15^. In addition to the mitochondrial respiratory chain, our findings draw attention to a potentially greater role of QCR6 in altering the KIC5 mutant phenotype.

**Table 1:** Top differentially expressed genes associated with mitochondrial and nuclear metabolism gene ontologies in the KIC5 mutant (Supplementary Table S10).

Gene ID	Annotation
**Mitochondrial metabolism genes of interest**	
PF3D7_0523300	Cytochrome *c* oxidase subunit ApiCOX18, putative
PF3D7_0622600	Cytochrome *b*–*c*1 complex subunit 9, putative
PF3D7_0817800	Conserved Plasmodium protein, unknown function
PF3D7_0823700	Mitochondrial import receptor subunit TOM7, putative
PF3D7_1022900	CDGSH iron–sulfur domain-containing protein, putative
PF3D7_1361700	Cytochrome *c* oxidase subunit 2A, putative
PF3D7_1426900	Cytochrome *b*–*c*1 complex subunit 6, putative
**Nuclear compartment genes of interest**	
PF3D7_0205900	26S proteasome regulatory subunit RPN1, putative
PF3D7_0318200	DNA-directed RNA polymerase II subunit RPB1
PF3D7_0405400	Pre-mRNA-processing-splicing factor 8, putative
PF3D7_0512200	Glutathione synthetase
PF3D7_0523000	Multidrug resistance protein 1
PF3D7_0802000	Glutamate dehydrogenase, putative
PF3D7_0831700	Heat shock protein 70
PF3D7_0903400	ATP-dependent RNA helicase DDX60, putative
PF3D7_0934100	TFIIH basal transcription factor complex helicase XPD subunit
PF3D7_1009400	Zinc finger protein, putative
PF3D7_1108700	Heat shock protein J2
PF3D7_1116800	Heat shock protein 101
PF3D7_1218300	AP-2 complex subunit mu
PF3D7_1219100	Clathrin heavy chain, putative
PF3D7_1227100	DNA helicase 60
PF3D7_1308200	Carbamoyl phosphate synthetase
PF3D7_1311800	M1-family alanyl aminopeptidase
PF3D7_1466300	26S proteasome regulatory subunit RPN2, putative

We next analyzed expression of genes associated with mitochondrial electron transport using gene sets derived from the Malaria Parasite Metabolic Pathways database (https://mpmp.huji.ac.il/maps/mitochondrionef.html). We observed a similar pattern of dysregulation to QCR6 at 6 hpi, 24 hpi, and 36 hpi (Fig. 5c, Supplementary Tables S10 and S13). Further supporting this is our additional analysis of gene sets associated with mitochondrial cytochrome *c* pathway expression (https://mpmp.huji.ac.il/maps/biogen_cytc.html), where we again observed a similar pattern of differential expression (Fig. 5d, Supplementary Table S13). Overall, we conclude the expressional changes to mitochondrial pathways in the KIC5 mutant are due to dysregulation of the mitochondrial electron transport chain.

### KIC5 disruption is associated with differential expression of nuclear metabolic pathways

**Figure 6:**
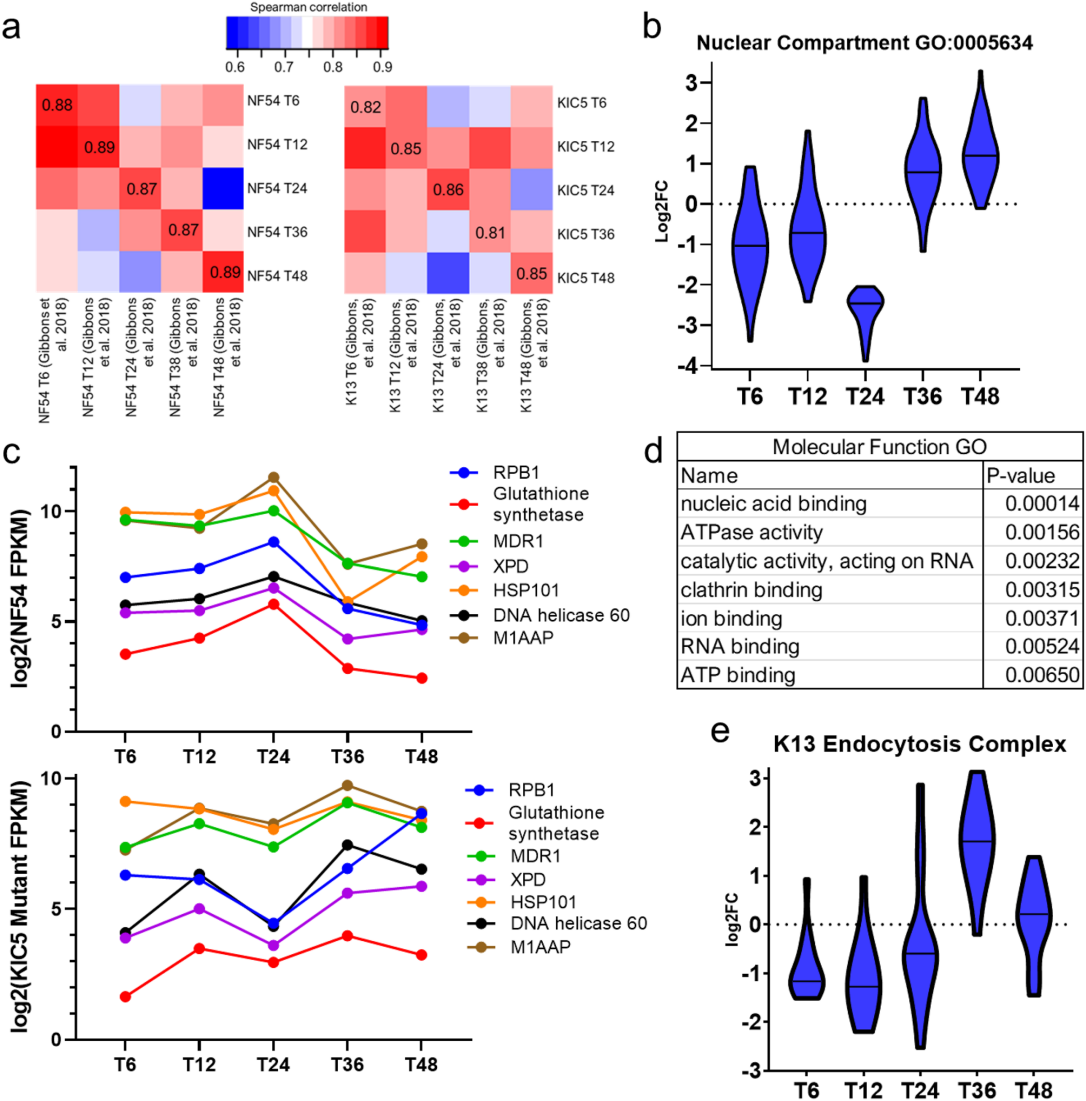
Nuclear metabolic activity is altered due to KIC5 disruption. We identified several similarities between the transcriptome of the K13 mutant, associated with altered DNA replication and repair expression, and the KIC5 mutant. (**a**) Correlation of the NF54 (left) and KIC5 mutant (right) datasets from this study to the WT and K13 mutant datasets from Gibbons et al.^14^ (Supplementary Fig. 5). Heatmaps made in R Studio using the pheatmap function and dataset correlation shown via Spearman correlation (Spearman correlation values available in Supplementary Table S8). Gene ontology enrichment analysis of KIC5 mutant expression across the IDC identified differential expression of genes associated with nuclear compartment (GO:0005634) (Supplementary Table S10). Log2-fold change (KIC5 mutant/NF54) analysis via violin plot (**b**) and FPKM analysis of expression in WT (top panel) and mutant (bottom panel) (**c**) shows dysregulation of these genes associated with nuclear compartment GO at 6 hpi, 12 hpi, and 24 hpi. (**d**) Molecular function GO enrichment of nuclear compartment (GO:0005634) genes in the KIC5 mutant. Log2-fold change of genes located in the K13 endocytosis complex shows a similar differential expression to nuclear compartment associated genes, further supporting the link between KIC5, K13, and the K13 complex with altered DNA replication and repair associated pathways (Supplementary Table S14). Log2-fold change data shown for the KIC5 mutant compared to WT expression and graphed on GraphPad Prism 9.

Because KIC5 has been functionally linked to K13, in addition to similar dysregulation patterns of the KIC5 and K13 mutants, we correlated our KIC5 mutant transcriptome to the transcriptome of the K13 *piggyBac* mutant (Fig. 6a, Supplementary Fig. 5, Supplementary Table S8)^14^. K13 and KIC5 mutant transcriptomes were highly correlated at 24 hpi and at 48 hpi. As both genes are dysregulated at 24 hpi in their respective mutants, this suggests similar transcriptomic signatures at these stages of development.

Chemogenomic profiling and transcriptomic analysis of the K13 mutant linked K13 to nuclear metabolism via an antagonistic relationship between DNA replication and repair pathway expression and K13 expression^13,14^. In the KIC5 mutant, we identified downregulation of genes associated with the nuclear cellular compartment (Fig. 6b). These include genes associated with various direct and indirect pathways of the nuclear metabolism: DNA replication, repair, or binding (DNA helicase [PF3D7_1227100], TFIIH basal transcription factor complex helicase XPD subunit [PF3D7_0934100], and zinc finger protein, putative [PF3D7_1009400]), RNA/mRNA metabolism (pre-mRNA-processing-splicing factor 8, putative [PF3D7_0405400] and ATP-dependent RNA helicase DDX60, putative [PF3D7_0903400]), clathrin binding (clathrin heavy chain [PF3D7_1219100] and AP2 complex subunit mu [PF3D7_1218300]), heat shock response (HSP70 [PF3D7_0831700] and HSPJ2 [PF3D7_1108700]), and the PTEX complex (HSP101 [PF3D7_1116800]) (Table 1, Supplementary Table S10). These genes are downregulated at 24 hpi, the same timepoint of KIC5 upregulation, and a similar pattern of dysregulation in the K13 mutant (Figs. 2a, 6b). Comparison of FPKM expression in the KIC5 mutant vs the isogenic wild-type NF54 supports these findings, with a decrease in expression of these genes at 24 hpi (Fig. 6c). GO molecular function revealed enrichment of nucleic acid binding, ATPase activity, catalytic activity acting on RNA, and ion binding (Fig. 6d). Considering the putative interactions and domains of the KIC5 protein in *P. falciparum* (Fig. 1a, Supplementary Fig. 2b,c), these findings support the role of KIC5 function in nuclear metabolism, especially in early ring-stage responses to oxidative stress. Lastly, gene expression analysis of the K13 endocytosis complex show a similar pattern of dysregulation to *nucleus* GO term genes (downregulation at 6–24 hpi, and upregulation at 36 hpi), thereby strengthening the link between KIC5, K13, and nuclear metabolism (Fig. 6e, Supplementary Table S14).

## Discussion

*P. falciparum* has a tightly controlled and synchronized pattern of gene expression, and alterations to this pattern can yield a variety of phenotypic effects that can alter the parasite’s response to external stressors^20^. Our study presents the first in-depth analysis of KIC5 and its roles in altering the transcriptome and ART-S response of *P. falciparum*. A critical component of this study included validating and correlating our findings to established ART-S transcriptomic patterns while identifying novel expression patterns associated with KIC5 disruption. We confirmed that changes to the KIC5 mutant transcriptome were not due to morphological shifts in IDC development or RNAseq sample stage distribution, establishing the single-insertion disruption of KIC5 to be the primary cause of dysregulation of the mutant transcriptome. Additionally, we found that housekeeping pathway expression was highly correlated between our NF54 and KIC5 samples, supporting transcriptional alignment. Furthermore, we found high correlations between our isogenic wild-type NF54 clone and other previously studied NF54 transcriptome, in addition to observing correlations between the KIC5 mutant and the K13 mutant at 24 hpi, alluding to a multitude of similarities between both transcriptomes^14^.

We identified a shift in KIC5 expression in WT from 6 to 24 hpi in the mutant. Attention in this study was subsequently given to identify pathways significantly differentially expressed at three timepoints of interest, 6 hpi, 24 hpi, and 36 hpi. These timepoints exhibited more differentially expressed genes compared to 12 hpi and 48 hpi, when mitochondrial metabolism and nuclear metabolism pathways identified significant were differentially expressed. Recent studies have been published investigating how the mitochondria and its energy production and redox system plays a role in recognizing and regulating ART response in *P. falciparum*^25,26^. We observed differential expression in the mitochondrial electron transport chain in the KIC5 mutant at 6 hpi, 24 hpi, and 36 hpi, supporting this hypothesis that suggests KIC5 is a downstream regulator of mitochondrial activity. Mutations to K13 have been linked to altered DNA replication and repair metabolism and, with KIC5 interacting directly with K13 via the K13 endocytosis complex, we observed disruptions to DNA metabolism in the KIC5 mutant. DNA replication, DNA repair, mRNA processing, and transcriptional factors were found to be downregulated at early ring to early trophozoite stage and upregulated at late trophozoite stages. Because of this, we hypothesize any ART-mediated damage to DNA, nuclear proteins, or associated downstream pathways are not resolved at 6 hpi and 24 hpi in the KIC5 mutant, thereby weakening the parasite’s ART response. Additionally, because genes of the K13 endocytosis complex are also similarly dysregulated in the KIC5 mutant, our data supports K13, and now links KIC5, as regulatory elements in homeostatic nuclear metabolism.

**Figure 7:**
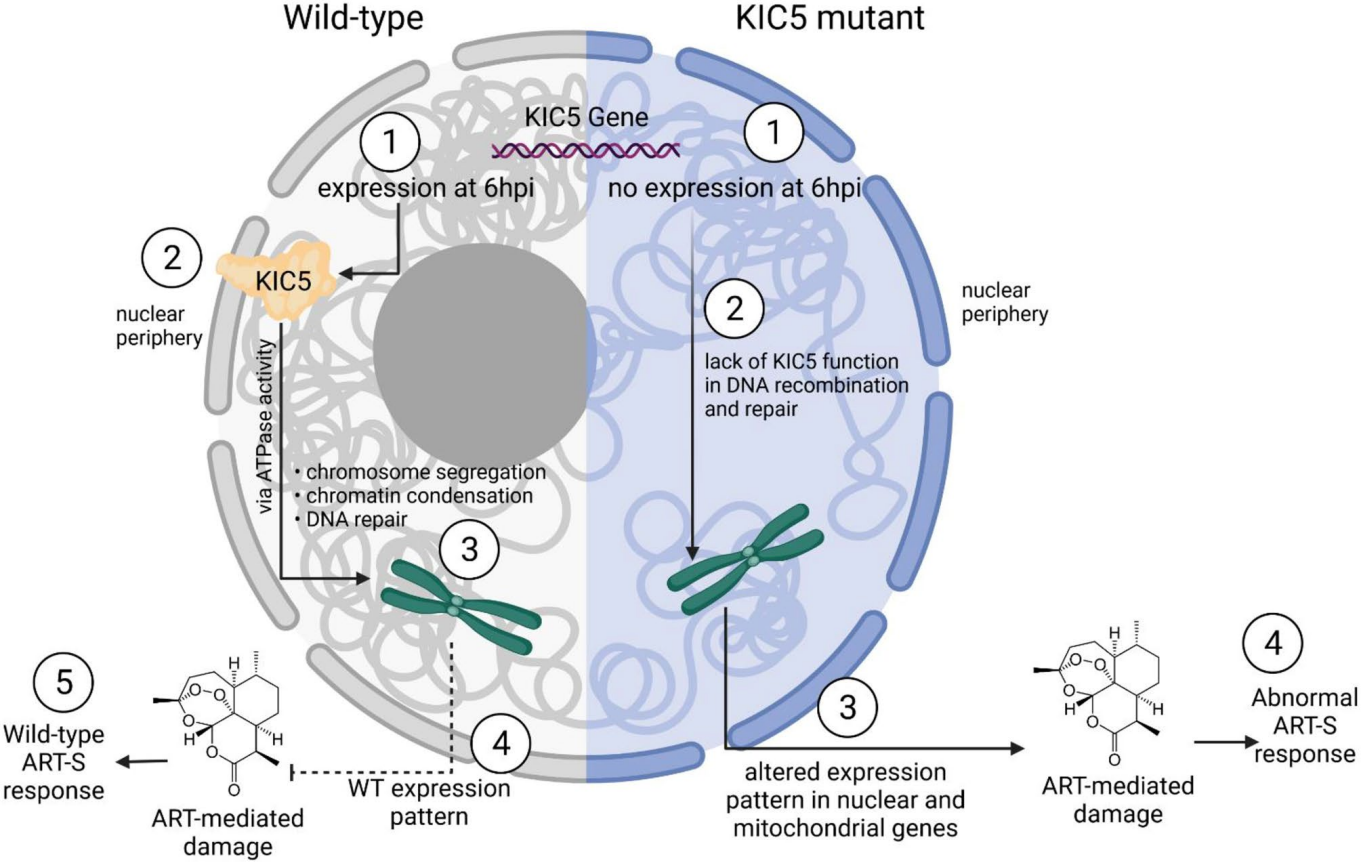
Mechanism of KIC5 mutant sensitivity to artemisinin due to the putative activity of the KIC5 protein. In the isogenic wild-type NF54 clone, KIC5 expression at 6 hpi leads to the gene’s activity in chromosome segregation, chromatin condensation, and DNA repair during ring stage, a critical period of artemisinin resistance in *P. falciparum*. KIC5 activity leads to wild-type expression pattern of stress response mechanisms under artemisinin exposure, leading to wild-type artemisinin sensitive phenotype. In the KIC5 mutant, however, dysregulation of KIC5 expression from 6 to 24 hpi leads to a lack of KIC5 protein activity during ring stage. Temporal changes to KIC5 activity led to altered expression of genes linked to artemisinin stress response, including various nuclear and mitochondrial pathways. Under artemisinin exposure, altered expression patterns lead to abnormal artemisinin response and increased sensitivity. Created with BioRender.com.

Although additional studies are needed to determine complete functional processes of KIC5, we know that this gene is associated with the K13 endocytosis complex and has putative ATPase domains linked to DNA repair^16^. While KIC5 is dispensable under normal culturing conditions, our findings point to KIC5 being essential under ART exposure at 6 hpi as a regulator of homeostatic nuclear activity, with dysregulation of this gene altering stress response-related factors in the parasite, including nuclear metabolism, mitochondrial activity, and K13 endocytosis complex expression (Fig. 7)^15^. Imbalance of these stress response-related factors may alter the parasite’s ability to respond to ART-mediated stress, thereby leading to increased drug sensitivity in the KIC5 mutant (Fig. 7). While we show that disruption of KIC5 transcriptionally alters ART stress-response related genes and pathways, we hypothesize changes in KIC5 activity at the protein level due to the significant temporal dysregulation in peak mRNA presence from 6 hpi in the NF54 parent clone to 24 hpi in the KIC5 mutant. Previously published findings by Le Roch et al. have shown a moderately high positive relationship between mRNA abundance and protein abundance during asexual blood stage, with genes of related functions showing similar mRNA and protein accumulation patterns^29^. Of the significantly differentially expressed genes identified in the KIC5 mutant, we found an overlap of 210 DEGs with mRNA/protein abundance correlation data present in the Le Roch et al. dataset (Supplementary Table S15)^29^. Of those 210 genes, the majority of genes (128 genes) showed positive correlation between mRNA and protein abundance during asexual blood stages. This includes several highlighted genes associated with nuclear cell compartment GO term in the KIC5 mutant, including clathrin heavy chain, heat shock protein 101, DNA-directed RNA polymerase II subunit RPB1, and 26S proteasome regulatory subunit RPN1 (Table 1). The direct correlation between transcript and protein abundance of several genes in WT leads to our hypothesis that the notable ~ 18 h shift in peak KIC5 mRNA abundance from 6 h in NF54 to 24 h in the mutant may temporally affect KIC5 protein activity. Additional studies are needed to identify the molecular targets and protein level dysregulation of this gene both under ideal conditions and under ART exposure.

With ART resistance emerging in Africa, there is a pressing need to comprehensively understand the relationship between *P. falciparum* and ART activity^1,30^. Here, we present the first characterization of how KIC5 dysregulation can alter the transcriptome of *P. falciparum* and lead to increased ART-S. The findings provided in this study connects KIC5’s function to nuclear DNA metabolism and reveals its downstream effect on multidrug resistance proteins, the mitochondrial electron transport chain, and the K13 endocytosis complex. Our study highlights the link between KIC5 and the parasite’s early ring-stage response to ART exposure, thereby establishing KIC5 as a novel regulator of ART-S and a tool for future investigations to address ART resistance.

## Methods

### Parasite culturing

*P. falciparum* parasites were frozen, thawed, and cultured using standard in vitro culturing protocols^31^. Parasites were maintained at 4% hematocrit in human O+ RBCs and culture media containing RPMI 1640 supplemented with Albumax II, hypoxanthine, HEPES, sodium bicarbonate, and gentamycin. Human O+ red blood cells were obtained from a commercial supplier, Interstate Blood Bank, Inc. A 37 °C mixed gas incubator was used with O_2_, CO_2_, and N_2_. NF54 and the KIC5 mutant were reported in our previous chemogenomic profiling study^13^.

### Intraerythrocytic developmental cycle growth analysis

The KIC5 *piggyBac* mutant was characterized for altered cycle progression compared to the isogenic wild-type NF54 parent clone by analyzing percentages of ring, trophozoite, and schizont stages during the 48-h life cycle via microscopy. KIC5 mutant and NF54 clone were thawed using standard protocols^31^. Both clones were similarly synchronized at the same time using 10% sorbitol for ring stage enrichment. Cultures were allowed to reach ~ 3% majority ring and were treated for the first time with sorbitol for 30 min and washed 3 × with warm RPMI and returned to culture. Sorbitol synchronization was performed when the cultures reached ~ 12 hpi at the next consecutive cycle, and again about 8 h later (~ 20 hpi) to obtain highly synchronized late rings. When the NF54 culture was determined to have reached 0 hpi (about half schizont to half rings), microscopy slides were taken for both NF54 and the KIC5 mutant cultures every 4 h for 48 consecutive hours, with culture media being replenished when needed. All assay slides were counted to determine parasitemia and percentages of ring, trophozoite, and schizont per slide (dividing the total number of rings, trophozoite, or schizonts counted by the total number of parasites counted, multiplied by 100 to get a percentage of each stage). Assay was performed with two bio-replicates and slides were counted twice. Data was further analyzed using Excel Spreadsheet. GraphPad Prism 9.3.1 was used to generate graphs and RStudio v2021.09.0 was used to perform Fisher’s Exact Test of microscopy counts of each cell cycle stage to determine significant difference between ring, trophozoite, and schizont values per timepoint for the KIC5 mutant vs NF54 clones (significance p-value < 0.05) (Fig. 1b, Supplementary Table S1).

### RNA sequencing

Total RNA samples were isolated at five timepoints post merozoite invasion of erythrocytic cells during the 48-h erythrocytic lifespan: 6 h post invasion (hpi), 12 hpi, 24 hpi, 36 hpi, and 48 hpi. Parasite clones of the isogenic wild-type NF54 and *piggyBac* mutant of KIC5 were maintained via standard conditions in a 200 mL culture at 4% hematocrit and ~ 5% parasitemia. Tightly synchronous parasite cultures were obtained via three rounds of sorbitol synchronization for ring stage isolation, followed by one round of Percoll synchronization for schizont stage isolation. Following one 48-h cycle for parasite culture recovery, microscopic analysis was used to identify 0 hpi, characterized by a population of roughly half late schizont stage parasites to half early ring stage parasites. Samples were harvested at the appropriate time point and slides analyzed for morphological stages (ring, early trophozoite, trophozoite, late trophozoite, early schizont, schizont, late schizont) were performed and analyzed similar to growth screen analysis via Excel, GraphPad Prism, and Fisher’s Exact Test of microscopy cell counts in R (Supplementary Table S2). RNA extraction from crude saponin-lysed samples was conducted using standard TRIzol extraction. Briefly, parasite cultures were transferred from culture flasks to conical tubes, centrifuged at 1750 rpm for 3 min, and the supernatant was removed. Infected RBC pellets were resuspended in cold 1 × PBS and red blood cells were lysed via saponin. After washing three times with cold PBS, the parasite pellet was resuspended in 1 mL TRIzol for every 50 mL of culture and stored in − 80 °C for RNA extraction. For RNA extraction, samples were allowed to reach RT and 2 μL of glycogen was added. The samples were incubated at 4 °C overnight, removed, and allowed to reach RT. After adding chloroform, the samples were vortexed vigorously and incubated at RT for 5 min. The samples were spun down at 12,000×*g* at 4 °C for 10 min, supernatant discarded, and 1 mL of 75% ethanol was added. The samples were spun at 10,000×*g* for 5 min, supernatant discarded, and the RNA pellet was allowed to dry. The RNA pellet was dissolved in 30 μL of DEPC-treated water while being incubated at 55 °C for 10 min. RNA was quantified using a Qubit fluorometer RNA HS Assay kit and quality determined via RNA ScreenTape Analysis. Using the Illumina TruSeq Stranded mRNA Library Prep Kit, mRNA was sequenced using Illumina Nextseq Mid-output X5 kit at 300 cycles.

### Obtaining gene expression data

Determination of gene dysregulation was performed similar to previously described protocols^14^. Briefly, reads were aligned to the *P. falciparum* 3D7 reference clone via the HISAT2 (Hierarchical indexing for spliced alignment of transcripts) program and raw counts were obtained using the FeatureCounts read summarization program. Sample transcripts were assembled using Cufflinks and the expression level of each gene was normalized as FPKM (Fragments per kilobase per million mapped reads), calculated using Cuffnorm.

### Sample correlation assessment

For additional quality control, NF54 and KIC5 mutant RNAseq data were compared to sequencing data from our previously reported K13 *piggyBac* mutant study (Supplementary Fig. 5, Supplementary Table S8)^14^. Using R (version 4.1.1) and RStudio, FPKM values of genes from NF54 in this study and NF54 from the previous study were correlated to identify Spearman’s rank correlation coefficient^14^ (Supplementary Tables S3 and S8). A similar process was performed for the KIC5 mutant data in this study and the K13 *piggyBac* mutant data in the previously reported study (Supplementary Tables S3 and S8)^14^. Genes were disregarded unless expression data was present in samples from both studies. Spearman correlation coefficients were also determined for our KIC5 mutant and NF54 parent clone samples (Supplementary Table S5). Housekeeping pathway correlation assessment between NF54 and KIC5 mutant was performed via RStudio. Gene lists corresponding to each housekeeping pathway were obtained from the Malaria Parasite Metabolic Pathways database (https://mpmp.huji.ac.il/maps/all), and FPKM values in NF54 and KIC5 mutant were correlated via R (Supplementary Tables S6 and S7). Correlation values were plotted in a heatmap via heatmap.2 function in R. For analysis of mRNA and protein abundance correlation from the Le Roch et al. dataset, genes with significant differential expression at any of the five timepoints analyzed in KIC5 mutant were cross-referenced with mRNA and protein abundance correlation data, which were compiled in Supplementary Table S15^29^.

### DEseq analysis and determination of gene dysregulation

DEseq2 (version 1.34.0) was used to normalize, calculate fold change (KIC5 mutant/NF54), and assess significant differentially expressed genes (DEG). A log2FC > 1.4 and FDR corrected p-value < 0.05 determines the gene as significantly upregulated in the KIC5 mutant. An observed log2FC < − 1.4 and FDR corrected p-value < 0.05 determines the gene as significantly downregulated in the KIC5 mutant. DEseq analysis was performed in RStudio.

### Timepoint dysregulation and graphs

Generation of NF54 and KIC5 mutant heatmaps to display differential expression at the timepoints analyzed in this study was performed in RStudio using heatmap.2 of the gplots (v3.1.1) package. Hierarchical clustering of FPKM values of the NF54 and KIC5 mutant gene expression at the five timepoints sampled in this study was performed via complete linkage clustering to determine cluster similarity between the various timepoints for NF54 and KIC5 mutant, with clustering visualized via heatmap with a column dendrogram. Volcano plots to show timepoint dysregulation of genes were also analyzed and created in RStudio with the ggplot2 package. For line graphs, bar graphs, and violin plots (FPKM or log2foldchange values) of specific genes of interest or a collection of specific genes, the analysis and statistics were performed in GraphPad Prism 9.3.1.

### Identification and gene ontology enrichment of gene clusters of KIC5 mutant

RStudio was used to analyze and perform gene cluster analysis of KIC5 and NF54 expression data.

We measured the compactness of gene clustering in the KIC5 mutant using within-cluster sum of square (wss). We performed k-means clustering for KIC5 mutant gene fold change values (KIC5 mutant/NF54 WT) across all sampled timepoints. The total within-cluster sum of square (wss) was calculated for each k and plotted according to the number of clusters. Identification of the bend in the plot indicated appropriate number of clusters. After determining this number in RStudio, log2foldchange values for KIC5 mutant were input into RStudio and fold change heat map was generated via pheatmap (version 1.0.12) and hierarchal clustering. Gene ontology enrichment for each cluster was obtained using the PlasmoDB gene ontology analysis tool^32^.

### Gene ontology analysis of KIC5 mutant timepoints

Gene ontology enrichment analysis per timepoint for the KIC5 mutant was performed by testing GO-terms mapped to genes in the category of interest against a background of GO-terms mapped to all other genes in the analysis using our R package pfGO (v 1.1)^23^. Briefly, The GO-term database was created from the latest curated *P. falciparum* ontology available from PlasmoDB (accessed November 2021)^32^. GO terms are enriched in dysregulated gene categories in the KIC5 mutant vs. WT NF54 by timepoint and ontology. Terms “Up” and “Down” represent upregulation in the mutant compared to NF54 or downregulated in the mutant compared to NF54, respectively. Enrichment was assessed via weighted Fisher/elim-hybrid p-value ≤ 0.05^33^ (Supplementary Tables S9 and S10). Highlighted gene ontology enrichment terms at 6 hpi, 24 hpi, and 36 hpi are included in table format with volcano plot analysis (Fig. 3).

### Metabolic pathway analysis

Metabolic pathway lists were obtained from the Malaria Parasite Metabolic Pathways database (https://mpmp.huji.ac.il/maps/all) and downloaded (October 2021) onto Excel. For each metabolic pathway list, genes with their corresponding FPKM or log2-fold change values were extracted from combined lists of FPKM values (generated via Cuffnorm) or log2-fold change (mutant/WT) values (generated via DEseq) of genes in the KIC5 mutant across the sampled timepoints. Extracted log2-fold change values for genes in each pathway were used to create violin plots in GraphPad Prism 9.

### Bioinformatic analysis of KIC5 protein

NCBI Protein Blast (https://blast.ncbi.nlm.nih.gov/Blast.cgi) was used (February 2020) to determine conservation of KIC5 protein sequence across Apicomplexan species. Additionally, NCBI Protein Blast identified putative conserved protein domains in the KIC5 protein sequence. ExPasy Prosite Database along with Phobius Transmembrane Topology Predictor was used (February 2020) to identify putative localization signals, amino acid rich regions, along with other bioinformatically determined protein sequence characteristics^34,35^.

## Supplementary Information

Supplementary Tables.

Supplementary Figures.

## Data Availability

RNAseq data generated for this study have been deposited to the NCBI Gene Expression Omnibus (GEO) database with the accession number GSE205012. Processed RNAseq data are provided in Supplementary Data File S3 and S4. The reference IDC data was obtained from: Bozdech et al.^20^.
