# Supplementary material for: Protein KIC5 is a novel regulator of artemisinin stress response in the malaria parasite Plasmodium falciparum
Source: Sci Rep. 2023 Jan 9;13:399. doi: 10.1038/s41598-023-27417-6 (PMC9829687; doi:10.1038/s41598-023-27417-6)
Supplement: Supplementary file 2 — Supplementary Figures. [file 41598_2023_27417_MOESM2_ESM.docx]

**Protein KIC5 is a novel regulator of artemisinin stress response in the malaria parasite *Plasmodium falciparum***

Caroline Simmons^1,2^, Justin Gibbons^1^, Min Zhang^1^, Jenna Oberstaller^1^, Camilla Valente Pires^1^, Debora Casandra^1^, Chengqi Wang^1^, Andreas Seyfang^1,2^, Thomas D. Otto^3^, Julian C. Rayner^4^, John H. Adams^1^*

^1^ Center for Global Health and Infectious Diseases Research and USF Genomics Program, College of Public Health, University of South Florida, Tampa, Florida, USA.

^2^ Department of Molecular Medicine, Morsani College of Medicine, University of South Florida, Tampa, Florida, USA.

^3^Institute of Infection, Immunity, and Inflammation, College of Medical, Veterinary and Life Sciences, University of Glasgow, Glasgow, UK

^4^Cambridge Institute for Medical Research, University of Cambridge, Cambridge Biomedical Campus, Cambridge, UK.

Corresponding author email addresses: ja2@usf.edu


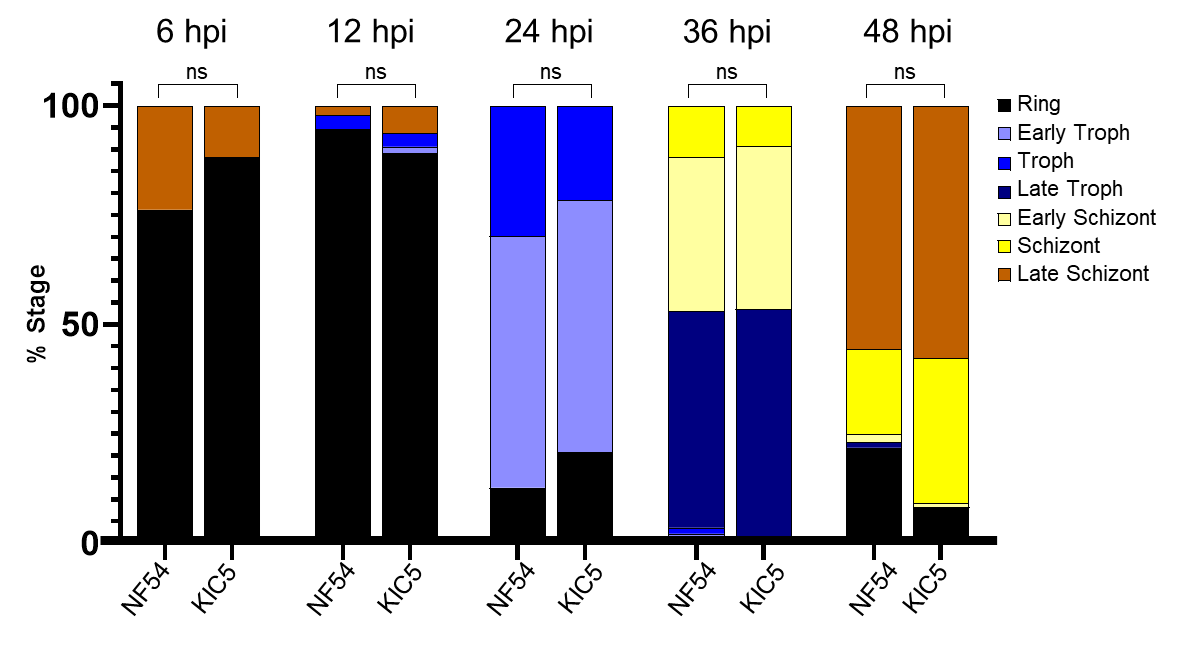


**Supplementary Figure 1.** Morphological stage comparison of KIC5 mutant RNAseq samples to WT NF54 RNAseq samples show no significant difference in cell cycle composition for each sample. Samples were analyzed for similarity via microscopy and statistical significance analyzed by Fisher’s Exact test of microscopy cell stage counts per timepoint sampled (Supplementary Data file S2).


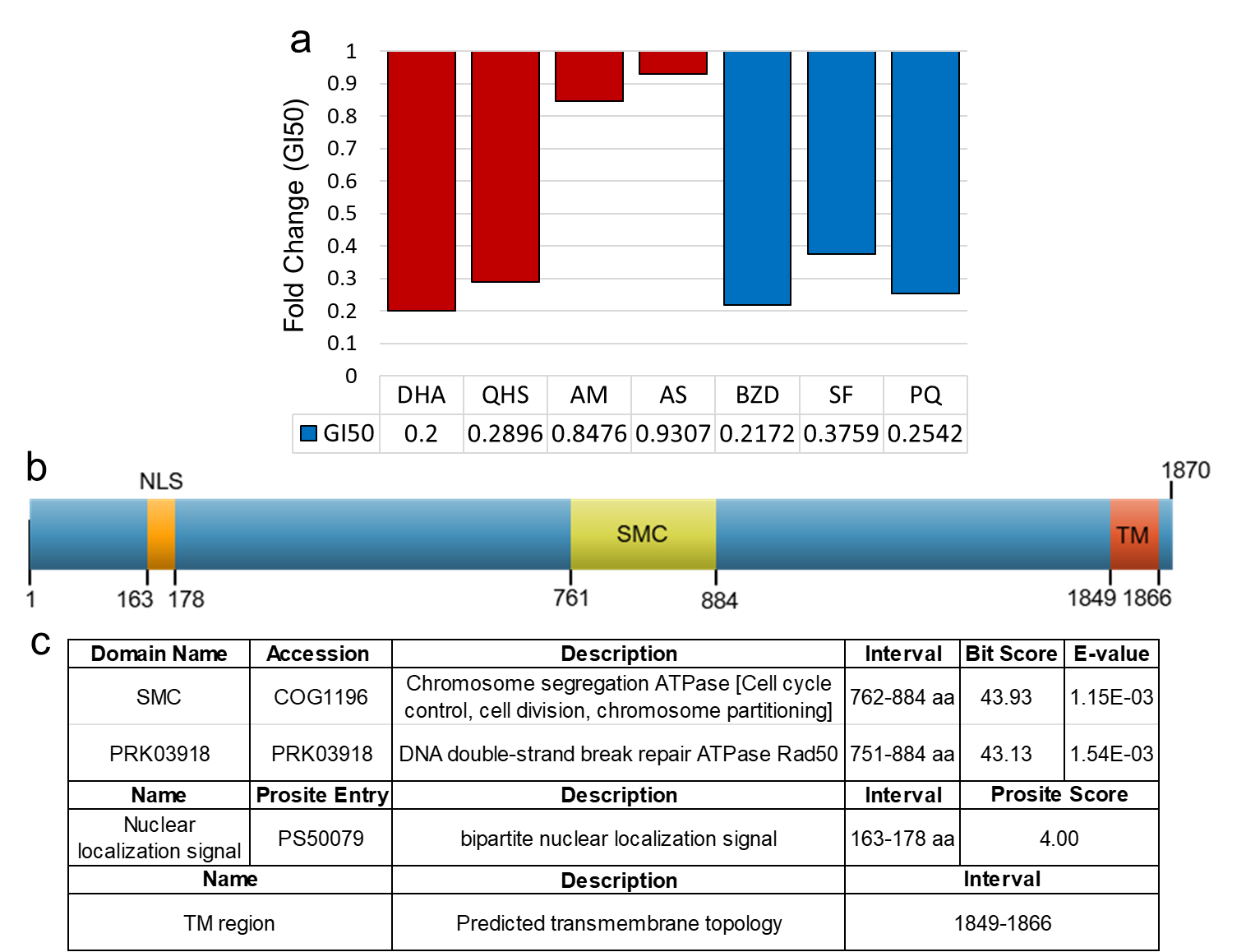


**Supplementary Figure 2.** (**a**) Fold change GI50 values of the KIC5 mutant to ART drugs (highlighted in red) previously assayed in (Pradhan, et al. 2015) show increased drug sensitivity. Also shown are fold change GI50 to BZD, SF, and PQ, all shown to have correlated drug response profiles to ART in previously performed chemogenomic studies (Pradhan, et al. 2015). (Abbreviations: DHA: dihydroartemisinin, QHS: artemisinin (qinghaosu), AM: artemether, AS: artesunate, BZD: benzimidazole, SF: sinefungin, PQ: primaquine) (**b**) KIC5 putative protein domain schematic shows a putative nuclear localization signal (NLS), SMC domain, and transmembrane region (TM). Additional information and significance of bioinformatically predicted domains shown in (**c**). Protein domains determine via NCBI Protein Blast (https://blast.ncbi.nlm.nih.gov/Blast.cgi), nuclear localization signal predicted via ExPasy Prosite database (https://prosite.expasy.org/), and transmembrane topology predicted via Stockholm Bioinformatics Centre Phobius (https://phobius.sbc.su.se/).

**
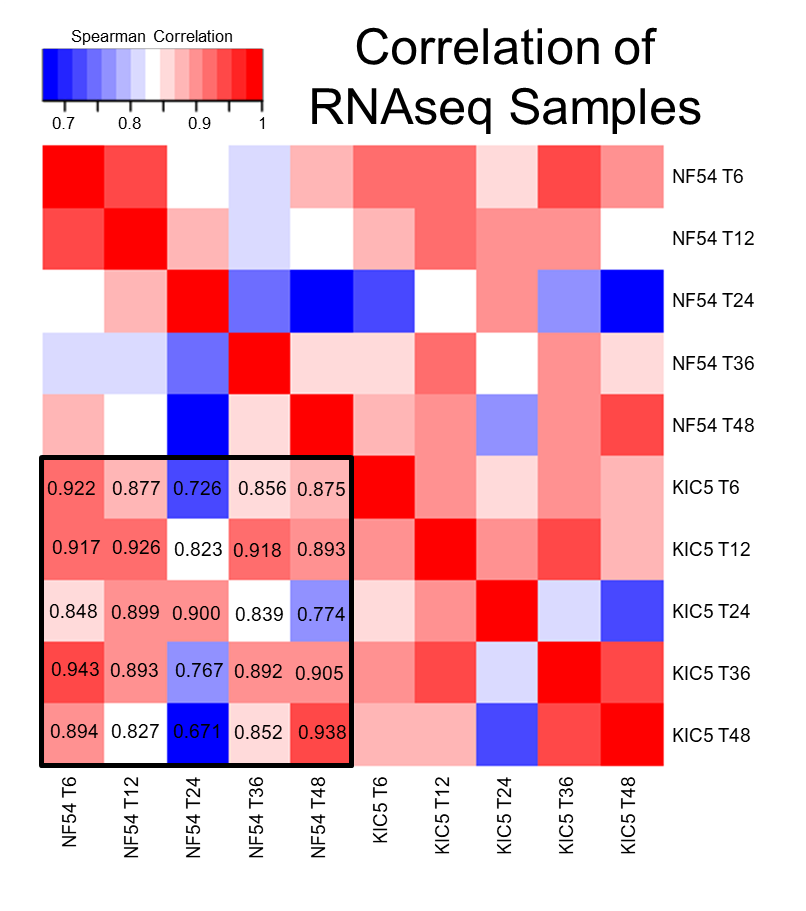
**

**Supplementary Figure 3**. Correlation between the NF54 parent clone and KIC5 mutant. Heatmap shows spearman correlation of NF54 to itself, the KIC5 mutant to itself, and NF54 and KIC5 mutant to each other (values in black box). All Spearman correlation values of FPKM expression obtained via R analysis and available in Supplementary Table S5. Heatmap made with heatmap.2 function in R.


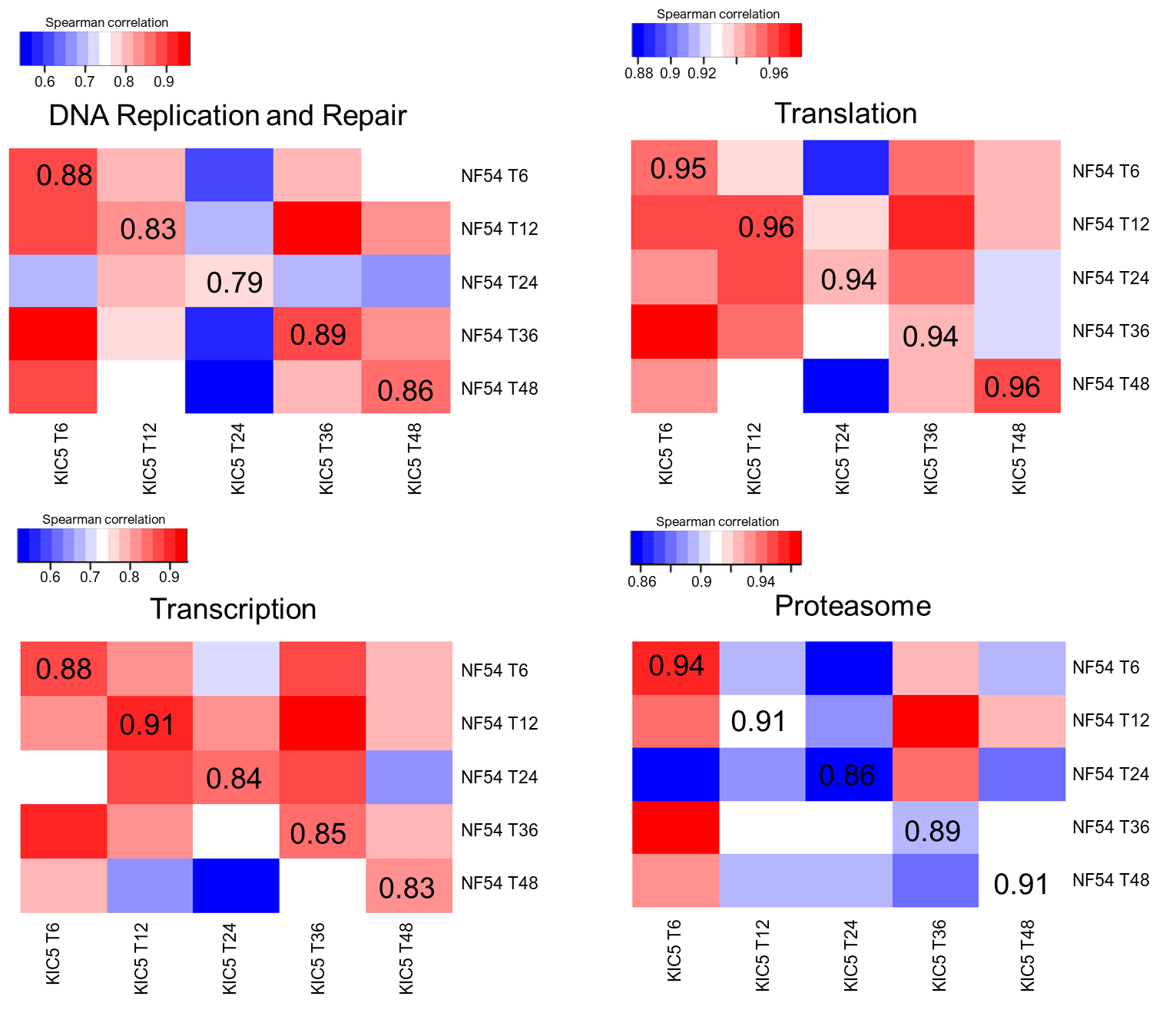


**Supplementary Figure 4**. Spearman correlation of housekeeping pathway FPKM gene expression between the NF54 clone and KIC5 mutant for transcriptional alignment analysis. Analysis shown for the following pathways at five timepoints during the intraerythrocytic development cycle: DNA Replication and Repair, Translation, Transcription, and genes of the Proteasome. Gene set IDs and FPKM expression shown in Supplementary Table S6, with correlation values shown in Supplementary Table S7. Spearman correlation values and heatmaps generated in R.


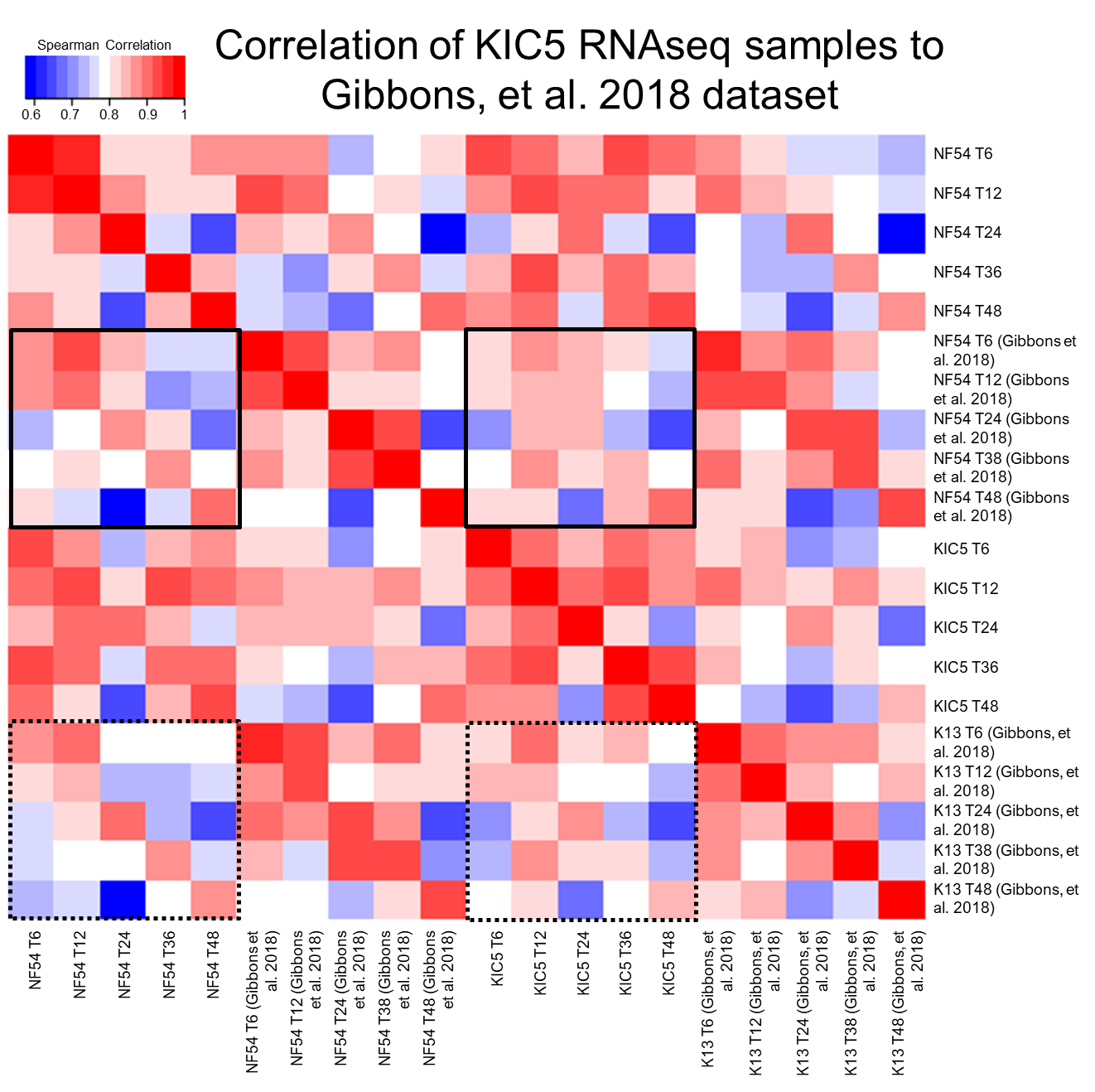


**Supplementary Figure 5**. Heatmap showing spearman correlation of the NF54 transcriptome from the previously published Gibbons, et al. 2018 study to the NF54 and KIC5 mutant samples from this study (solid-line boxes). Similar correlations were made between the published K13 *piggyBac* mutant transcriptome (Gibbons, et al. 2018) to our NF54 and KIC5 mutant datasets (dashed-line boxes). Spearman correlation values between all samples available in Supplementary Table S8 and generated in RStudio.


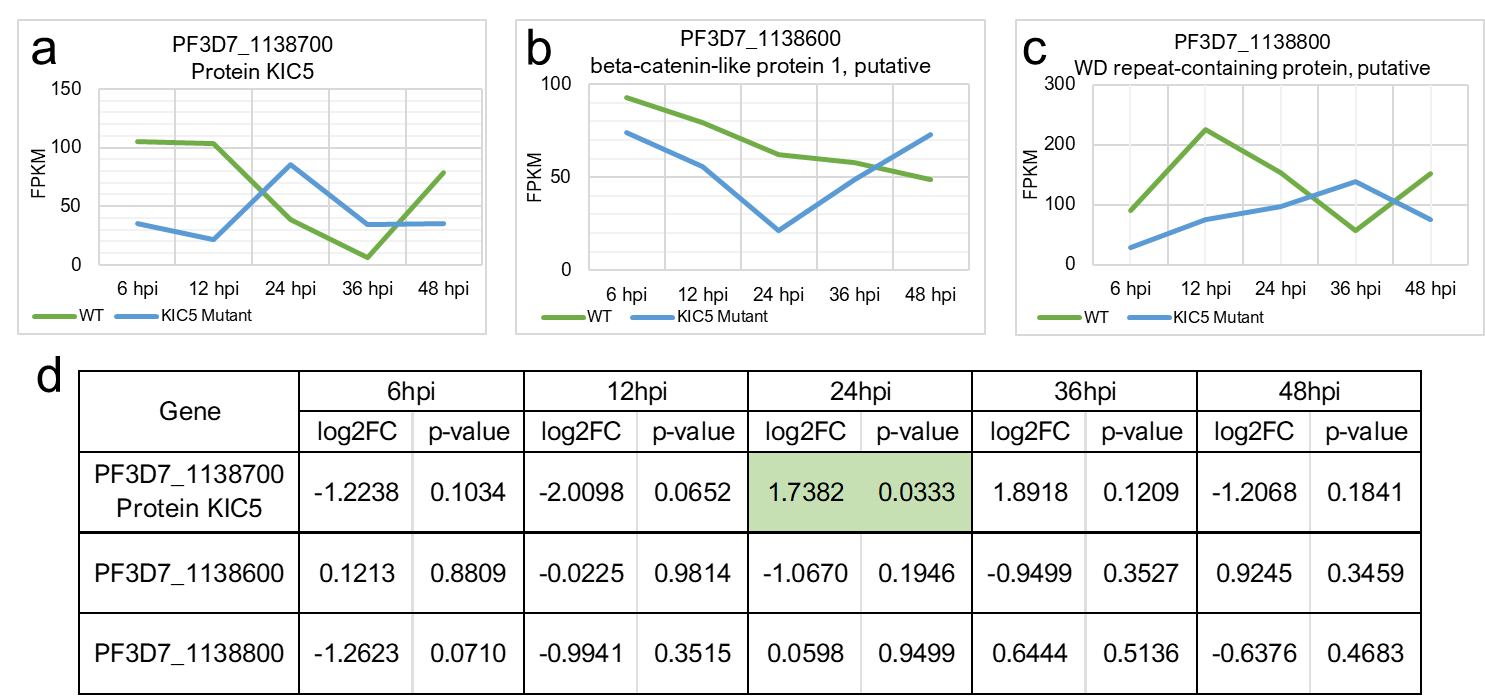


**Supplementary Figure 6.** FPKM expression in the NF54 WT (green line) and KIC5 mutant (blue line) of Protein KIC5 (**a**) and the two genes adjacent to KIC5 in the genome: beta-catenin-like protein 1 (**b**) and WD repeat-containing protein (**c**). Analysis of significant fold change of these genes across the five timepoints sampled in this study show significant gene upregulation of KIC5 at 24hpi (shaded green panels), with no significant gene dysregulation of the two genes adjacent to KIC5 at any timepoint tested (**d**).


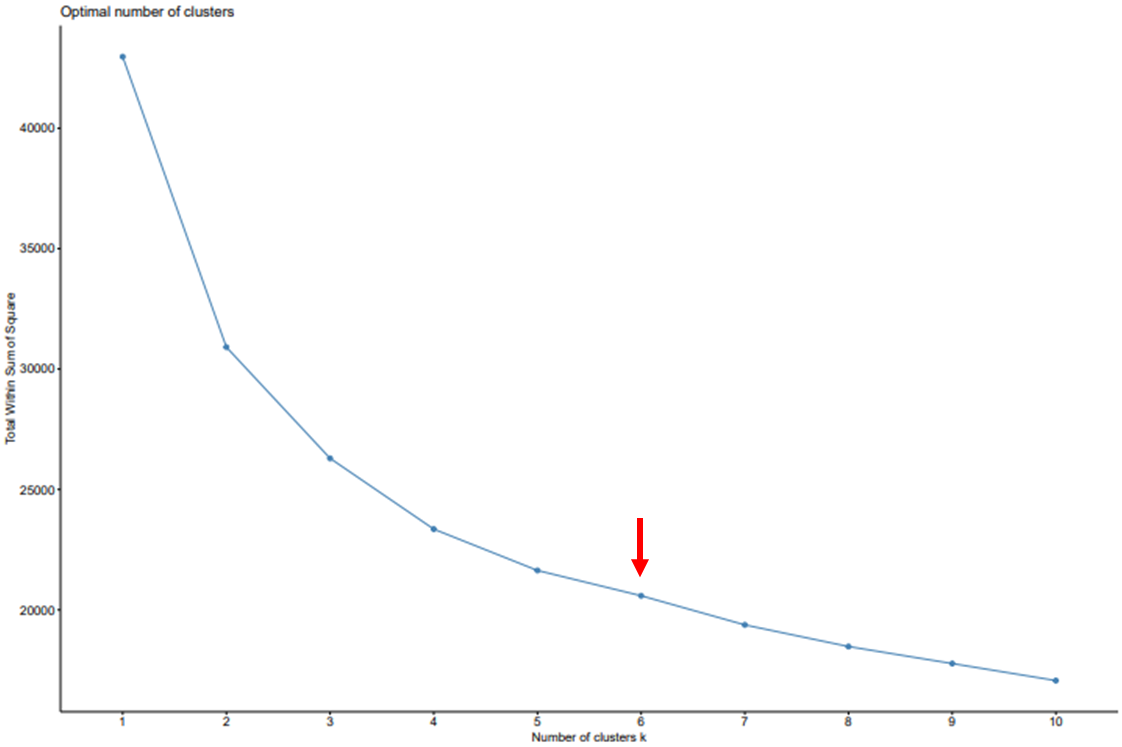


**Supplementary Figure** **7**. Plot showing the optimal number of clusters for gene clustering analysis of fold change expression in the KIC5 mutant. The total within-cluster sum of square (wss) was calculated and plotted for each k after k-means clustering for KIC5 mutant gene fold change values (KIC5 mutant/NF54 WT) across all sampled timepoints. Identification of the bend in the plot (red arrow) indicated appropriate number of clusters and guided subsequent gene clustering analysis.


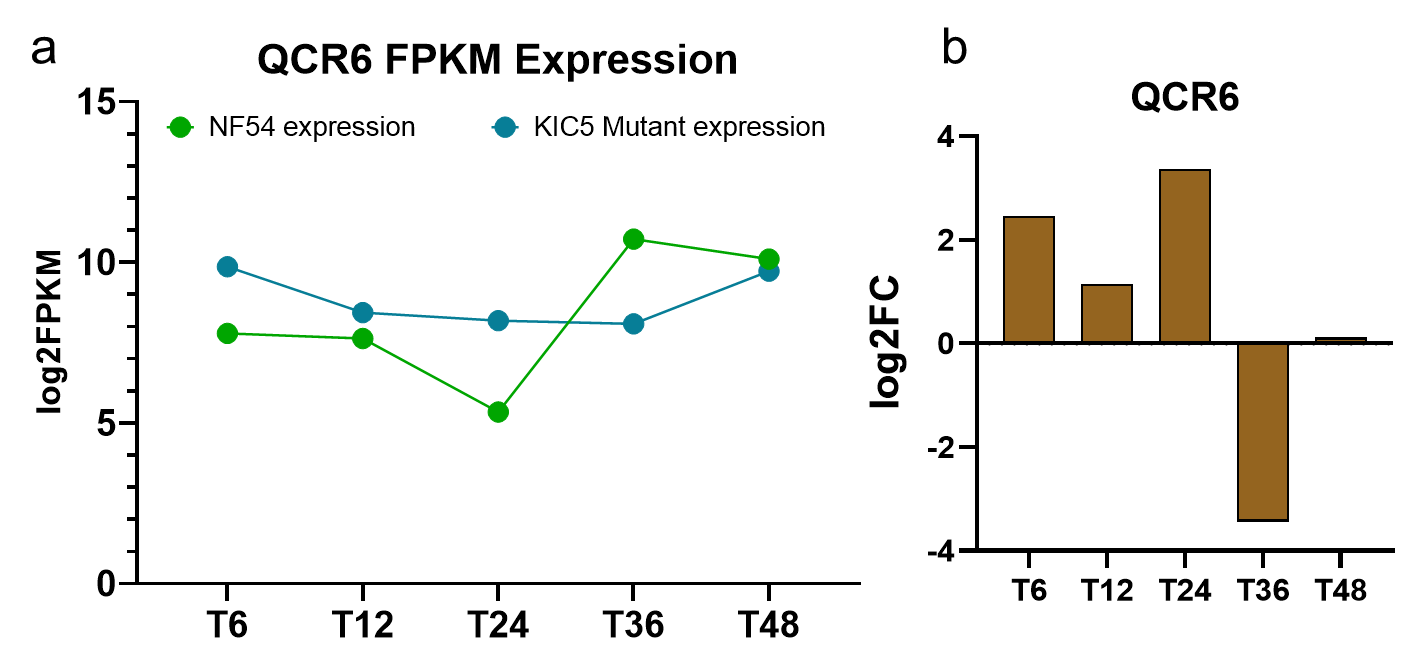


**Supplementary Figure 8.** (**a**) The QCR6 gene, associated with mitochondrial respiratory chain complex III [cytochrome b-c1 complex subunit 6, putative, (PF3D7_1426900)] demonstrates differential expression in the KIC5 mutant (blue line) compared to the WT NF54 strain (green line). (**b**) Fold change analysis of QCR6 expression in the KIC5 mutant compared to WT NF54 shows a pattern of expression consistent with mitochondrial ETC pathway dysregulation in the KIC5 mutant. FPKM values and fold change values shown via log2 transformation (Values available via Supplementary Data file S3 and S4, respectively).
